# Characterization and Preparation of Furanic-Glyoxal Foams

**DOI:** 10.3390/polym12030692

**Published:** 2020-03-20

**Authors:** Xuedong Xi, Antonio Pizzi, Hong Lei, Guanben Du, Xiaojian Zhou, Yuying Lin

**Affiliations:** 1 Yunnan key laboratory of wood adhesives and glue products, Southwest Forestry University, Kunming 650224, China; xuedong.xi@univ-lorraine.fr (X.X.); lfxgirl@163.com (H.L.); guanben@swfu.edu.cn (G.D.); xiaojianzhou1982@163.com (X.Z.); 2 ENSTIB-LERMAB, University of Lorraine, 27 rue Philippe Seguin, 88000 Epinal, France; 3 LERMAB, University of Lorraine, 88000 Epinal, France

**Keywords:** furfuryl alcohol, glyoxal, rigid foams, combustion properties, MALDI-ToF, FTIR

## Abstract

Synthetic foams have become an essential industrial product for a great variety of applications. Furfuryl alcohol, as a biomass chemical, was reacted with glyoxal at room temperature to prepare furanic-glyoxal rigid foams, and p-toluenesulfonic acid was used as a catalyst to initiate the reaction. Foams with different molar ratios (furfuryl alcohol/glyoxal) were prepared in this work, and uniform cells foams have been obtained. Their compression resistance, 24-h water absorption, density, and other basic properties were tested. Scanning electron microscopy (SEM) was used to observe the cellular morphology of the foams prepared, thermogravimetric analysis (TGA) helped to understand their thermal and combustion properties, and FTIR and Matrix Assisted Laser Desorption Ionisation Time of Flight (MALDI ToF) mass spectroscopy to explain the structure of the resulting foams to clarify the reactions occurring during foaming. The results show that the compression resistance of furanic-glyoxal foams declined as the furfuryl alcohol/glyoxal ratio decreases also. SEM observations revealed that foams with open-cell were obtained when furfuryl alcohol was added in greater amounts, and more closed cell structures were formed as the proportion of glyoxal increased. TGA results showed that the initial ignition temperature of furanic-glyoxal foams is ~200 °C higher than that of wood, and the smaller comprehensive combustion index S (about 0.15 × 10^−7^ (%^2^ K^−3^ min^−2^)) indicates that the foam burns slowly and has poor flammability, that is, it is not easy to burn.

## 1. Introduction

Foams are very widely used materials, due to their advantages of light weight, heat insulation, sound absorption, shock resistance, moisture resistance, corrosion resistance, etc. Therefore, they can be applied as thermal insulation materials, sound insulation materials, packaging materials, in industry, agriculture, construction, and transportation [1,2,3]. Since the advent of synthetic foams, their application has become progressively more extensive, and their variety has continued to increase. The most common traditional foams are mainly polyurethane (PUR) [3,4,5,6], polystyrene (PS) [7,8], polyvinyl chloride (PVC) [9], polyethylene (PE) [10,11], and phenolic resin-based (PF) foams [12]. In recent decades, as the research on biomass materials to replace petrochemical products has become a hot topic, the study of tannin and furfuryl alcohol as the main raw materials to prepare tannin-furan rigid foams has received much attention [13,14,15,16,17,18,19,20,21].

Tannins are natural polyphenolic substances used by plants to protect themselves from insects and fungi. They are widely distributed in the plant kingdom, and they have been used in many industrial applications such as wood adhesives and leather tanning [22]. Furfuryl alcohol is an important biosourced chemical raw material, which is mainly used in producing furan resins. In addition, it is also widely used in industrial production of dyes, synthetic fibers, rubber, pesticides, and casting. This chemical is usually obtained from hemicelluloses of different types from agricultural waste such as sawdust, wheat, or corn, and is therefore considered a natural product. Recently, there has been much interest in using furfuryl alcohol to prepare furanic polymer foams under acid catalysis. However, they presented many drawbacks, the work on them mainly been limited to analysis of their structure [23]. Moreover, a work using furfuryl alcohol and formaldehyde with wood flour as additives to prepare foams is on record, but the foams obtained did not have acceptable properties, and moreover toxic formaldehyde was used, thus yielding unsatisfactory results [24].

In the work presented here, furfuryl alcohol was reacted with glyoxal to prepare a furfuryl alcohol-glyoxal foam. Glyoxal is nontoxic and non-volatile compared with formaldehyde, and it has been widely used as a green environmental additive in the papermaking and textile industries [25]. The reaction between glyoxal and furfuryl alcohol is confirmed, and a study has already reported furfuryl alcohol-glyoxal resin as a wood adhesive applied in the preparation of plywood, with rather encouraging results being obtained [26]. However, there are no reports on the use of furfuryl glyoxal to produce rigid foams. In this work, furfuryl alcohol, glyoxal, and an appropriate amount of olive stone flour were mixed at room temperature, and p-toluene sulfonic acid was used as a catalyst to obtain a self-blowing rigid foam.

## 2. Materials and Methods

The glyoxal (C_2_H_4_O_2_, 40% water solution) used are of analytical grade (AR), obtained from Acros Organics (Ilkirch, France); furfuryl alcohol (AR) was obtained from Sigma-Aldrich (Saint Louis, France); p-Toluenesulfonic acid (AR) was obtained from Acros Organics (Geel, Belgium); and diethyl ether was obtained from Sigma-Aldrich (Saint Louis, France). Two-hundred-mesh olive stone flour was obtained by (RPC Superfos, Taastrup, Denmark).

### 2.1. Foams Preparation

**Figure 1:**
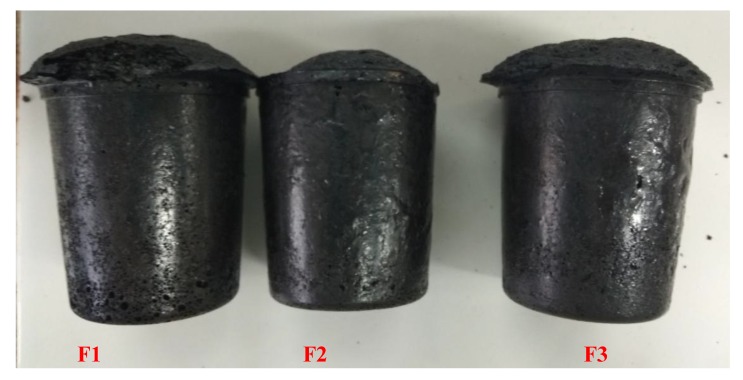
Picture of furanic-glyoxal foams prepared according to the formulations in Table 1.

Furfuryl alcohol, glyoxal, and olive powder, as shown in the Table 1, were mixed very well in a glass beaker, then p-toluene sulfonic acid (65% water solution) was added, the mixture was then manual stirred for 30 s, and then diethyl ether was added and mixed for another 10 s before the mixture was transferred to the foaming mold. The mixture was left in a fume hood at room temperature for ten minutes and then the foaming process took place. This foam was placed in ambient environment for 1 h, then it was dried in the oven at 103 °C for 4 h. The foam was removed from the foaming mold and placed at ambient temperature (25 °C and 12% relative humidity) for 2 days before its characterization. The foams prepared (Figure 1) were tested for their absorption by keeping them immersed in tap water at pH 7.2 at ambient temperature for two hours (Table 1).

### 2.2. SEM Observation

To investigate the cell microstructure and morphology of foams, scanning electron microscopy (Gemini SEM 300, Aalen, Germany) was used with an acceleration voltage of 10 kV. The mean cell size of foams were calculated by Nano Measurer 1.2 (Dept. of Chemistry, Fudan University, China) depending on the SEM images [27].

### 2.3. ATR FT-MIR Analyses

All of the sample extracts were analyzed with a PerkinElmer Frontier ATR-FT-MIR (Suzhou, China) provided by an ATR Miracle diamond crystal. The powder samples were laid on the diamond eye (1.8 mm) of the ATR equipment and the contact for the sample was ensured by tightly screwing the clamp device. Each extract was scanned registering the spectrum with 32 scans with a resolution of 4 cm^−1^ in the wavenumber range between 600 and 4000 cm^−1^.

### 2.4. MALDI-TOF Analysis

Samples for matrix-assisted laser desorption ionization time-of-flight (MALDI-TOF) analysis were prepared by first dissolving 7.5 mg of sample powder in 1 mL of a 50:50 *v*/*v* acetone/water solution. Then, 10 mg of this solution was added to 10 µL of a 2,5-dihydroxy benzoic acid (DHB) matrix. The locations dedicated to the samples on the analysis plaque were first covered with 2 µL of a NaCl solution 0.1 M in 2:1 *v*/*v* methanol/water, and pre-dried. Then, 1 µL of the sample solution was placed on its dedicated location and the plaque was dried again. Red phosphorous was used to standardize the MALDI equipment. MALDI-TOF spectra were obtained using an Axima-Performance mass spectrometer from Shimadzu Biotech (Kratos Analytical Shimadzu Europe Ltd., Manchester, UK) using a linear polarity-positive tuning mode. The measurements were carried out making 1000 profiles per sample with 2 shots accumulated per profile. The spectrum precision is of +1 Da.

### 2.5. Thermogravimetric Analysis (TGA)

To understand the thermal properties of the furanic-glyoxal foams, the thermogravimetric analysis (TGA) and derivative thermogravimetric (DTG) analysis were conducted using a thermogravimetric analyzer (TG, SDT Q600 TGA, TA Instruments, New Castle, USA). In each test, ~10 mg of foams was loaded in an alumina crucible. Then, air at a flow rate of 100 mL min^−1^ was individually used as carrier gas to analyze the combustion characteristics of the foam [28]. In each analysis, the sample was first heated from room temperature to 50 °C and then held for a certain period until the balance was steady. This provided a basis for sample analysis. Afterward, the sample was heated from 50 to 800 °C at a heating rate of 20 °C min^−1^.

### 2.6. 24-h Water Absorption

To investigate the water absorption of furanic-glyoxal foams and the effect of their densities on water absorption, 4 samples were tested for their 24-h water absorption.

### 2.7. Compression

The foams obtained by different formulations were compression tested. The tests were done under compression in an Instron 3300 dual column universal testing machine (Instron France, Elancourt, France) at a head rate of 1 mm/min.

## 3. Results and Discussions

### 3.1. Physical Properties of Furanic-Glyoxal Foams

To better describe the foaming process, some parameters are used to define the foaming behavior, such as (i) *t*_p_ (preparation time): the time from when the reaction mixture was placed in the foaming mold to when it started foaming, and (2) *T*_f_ (foaming temperature): the highest temperature reached during the foaming process. By comparing foams F1, F2, and F3 (Table 2), one can observe that the foaming temperature has decreased and the preparation time has increased, this being directly related to the proportion of furfuryl alcohol. From the foam formulations in Table 1, it can be clearly seen that the reason for foam F1 showing the highest foaming temperature and shorter preparation time is that the proportion of furfuryl alcohol is larger. Under acid catalysis, furfuryl alcohol self-condenses more rapidly and very exothermally generating a lot of heat. The apparent density of the foams prepared here is relatively similar, with only F2 being slightly higher, which can be explained by the smaller expansion height of foam F2 in Figure 1. As in F2, the expansion degree decreases this affects its compression performance. Foam F1 shows the largest 2 h water absorption, probably because it is composed of open-cells, the more detailed explanation for this being discussed in the SEM analysis section.

**Figure 2:**
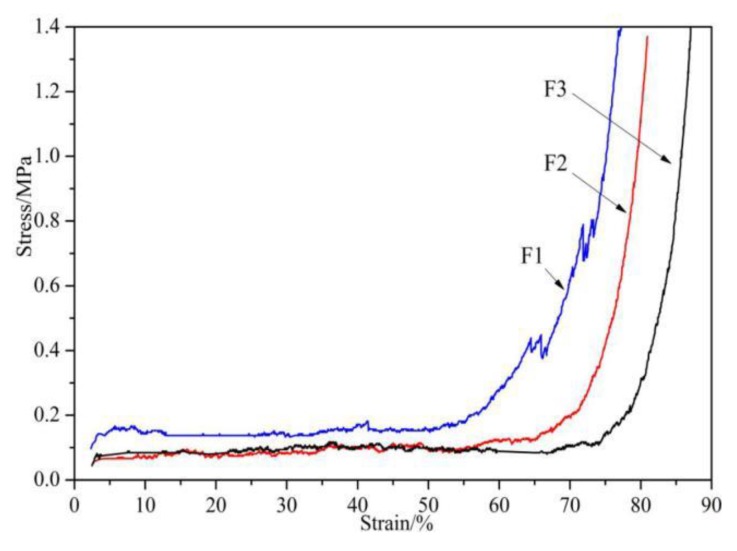
Compression of furanic-glyoxal foams.

The compression resistance of the foams was tested, and the results are presented in Figure 2. It can be observed that the compression curves of the foams can be divided into three stages: the elastic behavior of the foam’s cell wall bending, cell collapse, and densification [29]. The compression curve of Foam F1 is higher than that of the other two curves, which is mainly due to the difference in the relative proportion of the furfuryl alcohol used. Compared with F2 and F3 foams, the amount of furfuryl alcohol used in F1 is larger. As the proportion of polyfuran substances produced by the self-condensation of furfuryl alcohol is larger, F1 exhibits better hardness and compression resistance [30,31,32]. For the same reason, as the amount of glyoxal used increases, F3 shows the worst compressibility, as its densification process occurs the latest.

**Figure 3:**
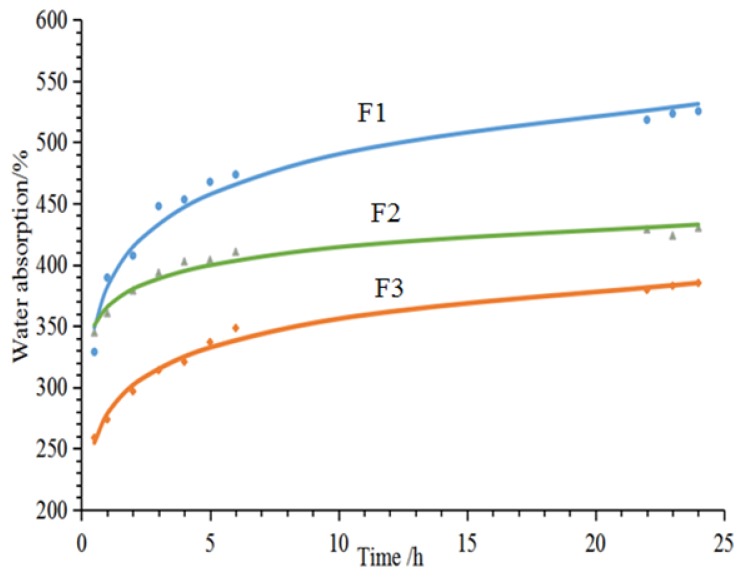
24-h water absorption of furanic-glyoxal foams.

Figure 3 shows the 24-h water absorption of furfuryl alcohol-glyoxal foams prepared from the formulations in Table 1. First, these three foams exhibit different levels of water absorption, which is directly related to their different cell morphologies. Foam F1 has the greatest water absorption, as it has open-cells, this being seen in the SEM results. For foam F2, although in the SEM analysis it appears that there are many pores in the cell walls, when looking closely, a thin film can be observed covering the pores. This means that the cells are blocked in foam F2, and not connected like the open-cells in the F1 foam. Therefore, foam F2 has the smallest water absorption. Furthermore, the water absorption levels markedly increases during the first five test hours, due to the storage of water in the cell cavity. Water absorption levels off after ~5 h of water immersion or just continues to increase at a very slower rate.

### 3.2. SEM Analysis

**Figure 4:**
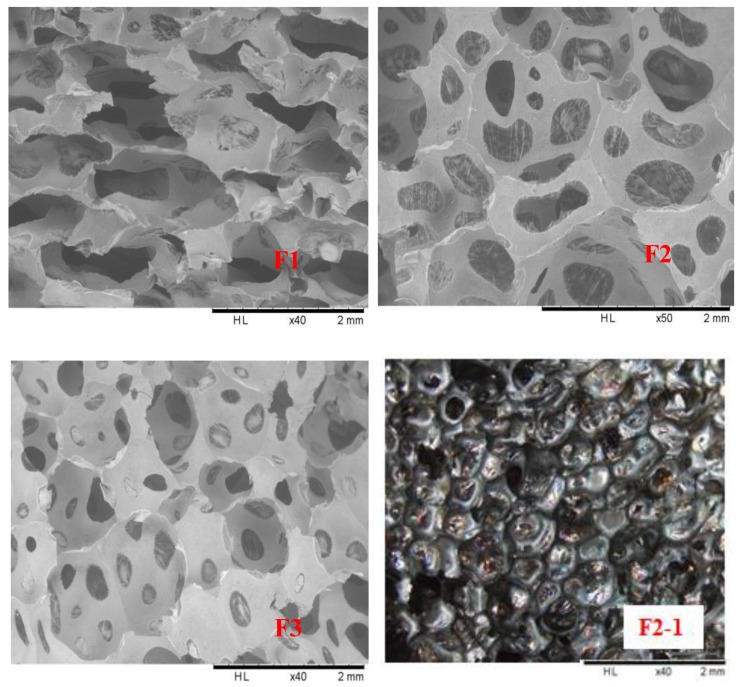
F1: SEM pictures of furanic-glyoxal foams (F1, F2, and F3) and microscope picture of F2 foam (F2-1).

Scanning electron microscopy (SEM) was used to observe the F1, F2, and F3 furanic-glyoxal foams cell structures, the results being shown in Figure 4. First, it is easy to see that foam F1 is composed of open-cells and that the cross-sectional shape of its cells is not regular, being of irregular geometric shapes, and not circular or polygonal. This is most likely due to the greater proportion of furfuryl alcohol used in F1. This leads to a more violently exothermal furfuryl alcohol self-condensation reaction. The higher intensity of F1 leads to a similar cell morphology as reported for furanic-lignin foams [33]. Second, the cell shape of foams F2 and F3 seems to be more spherical, and there is a large number of pores in the cell walls. In particular, the pores in the cell walls of foam F2 are significantly larger than in F3, and F3 is more similar to a closed cells foam. This seems to mean that as the amount of glyoxal increases in the formulation the foam cells will gradually change from being predominantly open-cells to being predominantly closed-cells. In addition, although there are many pores in the walls of F2 and F3 foam cells, a thin film on the pore can be observed at a closer inspection. This is confirmed by the light microscopy result as shown in the F2-1 (Figure 4). This film makes the two adjacent cells not completely connected, and this is the reason for foam F2 and F3 presenting a smaller water absorption than F1.

### 3.3. Thermogravimetric Analysis (TGA)

**Figure 5:**
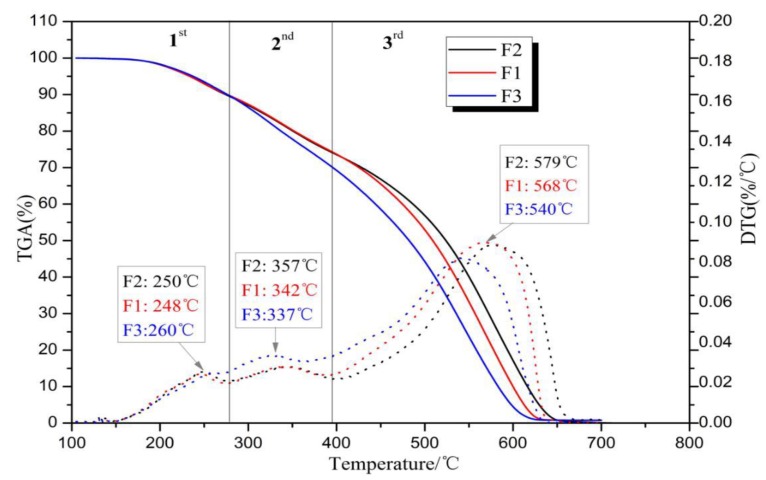
Thermogravimetric analysis (TGA) and derivative thermogravimetric (DTG) curves of foams in air atmosphere.

The TGA and DTG curves of the foams thermally degraded in air are shown in Figure 5, and their weight losses can roughly be divided into three stages: The first stage at temperatures before 360 °C is mainly due to the water removal and the volatilization and oxidation of light compounds in the furanic-glyoxal foams [34]. The main weight loss occurs in the third stage at temperatures in the 400 to 650 °C range, and 50% of weight loss occurs after 510 °C. This indicates that these foams have good heat resistance.

To identify the combustion characteristics of the foams, some parameters derived from TG–DTG curves were used to define their thermal behavior, such as *T*_i_ (initial temperature) representing the reaction initiation temperature; *T*_f_ (final or burnout temperature) being the temperature at which the reaction was complete and the mass loss remains nearly constant; *T*_max_ being the temperature where maximum mass loss occurs; maximum weight loss rate (*DTG*_max_) and its corresponding temperature (*T*_max_); and mean weight loss rate (*DTG*_mean_) [35]. All these parameters are recorded in Table 3. It is easy to observe that the initiation temperature of these three foams are very close, around 200 °C. This is higher than many reported lignocellulosic biomass, which are instead about 160–180 °C [36]. This indicates that furanic-glyoxal foams are not easy to ignite compared to wood or other lignocellulosic biomass. Furthermore, the combustibility characteristic index *S* of the foams is calculated [37,38] in accordance with the following formula.

(1) $$S=\frac{DTG\max\times DTGmean}{Ti^{2}\times Th}$$

**Figure 6:**
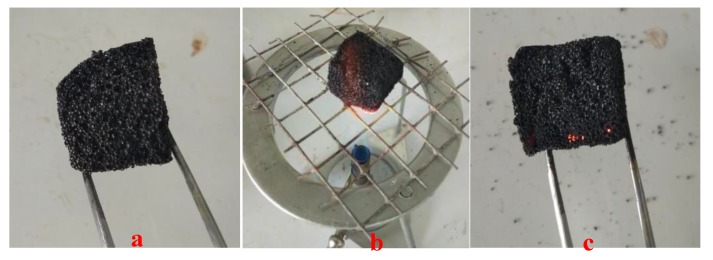
Pictures of ignition test of foam F2: (**a**) before ignition, (**b**) at ignition, and (**c**) after 5 min.

Physically, the higher the *S* value, the better the combustibility, and thus the material burns out faster. Conversely, a smaller *S* value indicates that the substance has poor flammability and burns slowly, that is, it is not easily combustible. In this work, the *S* value of the furanic-glyoxal foams is only ~0.15, which is far less than the *S* value of most lignocellulosic biomass (>5.89) [36]. This means that these foams have poor flammability and are not easily combustible, which is consistent with the result of ignition test in the laboratory according to literature methods [39]. In fact, as shown in Figure 6, this foam cannot be ignited. Even if the foams were placed on a Bunsen burner (around 600 °C) and heated for 5 min, they never ignited, and after being removed from the flame, they maintained their original appearance.

### 3.4. FTIR Analysis

**Figure 7:**
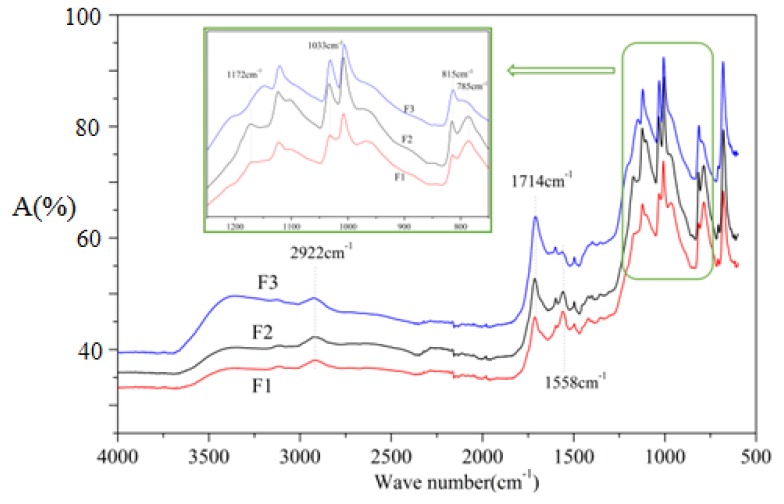
FTIR spectra of furanic-glyoxal foams without olive stone flour added.

To better explain the reactions occurring during the foaming process, foams F1, F2, and F3 were prepared according to the formulations in Table 1 without adding olive stone flour, and tested by FTIR, the results being shown in Figure 7. The wide peak at 3100–3500cm^−1^ is characteristic of the O–H stretching vibration [26,37,38], it can be observed from Figure 7 that curve F3 has a strong absorption peak here. This indicates that foam F3 contains more hydroxyl groups, which is mainly due to the larger amount of glyoxal used in the formulation. Some hydroxyl groups can be dehydrated to form carbon double bonds (–C=C–) under certain conditions, which can be observed at 1601 cm^−1^ [40]. Glyoxal reacts with furfuryl alcohol to generate hydroxyl groups, as will be further described in MALDI ToF analysis of the structures formed. The peak at 1558 cm^−1^ is attributed to C=C stretching vibration from the aromatic furan ring [26,41]. When comparing F1 and F3 (Figure 7) this peak is gradually less intense, indicating that the furan structure contained in the foam is decreasing, this being relative to the proportion of raw materials in the preparation formulation in Table 1. It is also apparent that the absorption peak of ether group observed at 1033 cm^−1^ is enhanced in the curves of F2 and F3 in respect to F1, which means that more ether bonds (C–O–C) are generated in foams F2 and F3. As more glyoxal was added to the preparation formulation of foams F2 and F3, ether bonds formed by the reaction of glyoxal with furfuryl alcohol. As in the F1 formulation, more furfuryl alcohol being added, the self-condensation under acidic conditions to produce methylene bridges is predominant. This is supported by the change in the intensity of the absorption peak at 2922 cm^−1^, as it belongs to the furan –CH_2_– stretching band [41].

### 3.5. MALDI ToF Analysis

**Figure 8:**
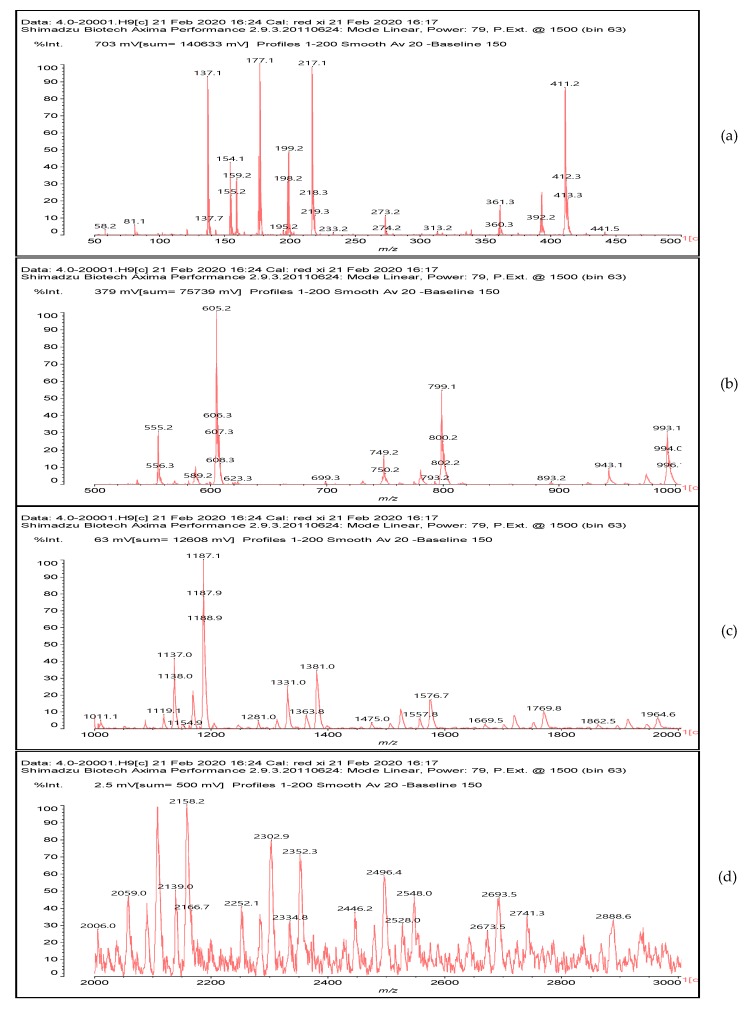
MALDI ToF spectrum of the furanic-glyoxal foams: (**a**) 50Da-500Da range. (**b**) 500Da-1000Da range. (**c**) 1000Da-2000Da range. (**d**) 2000Da-3000Da range.

To better analyze the chemical structures of these self-blowing foams and describe the progress of the reaction during the foaming process, a hardened foam F2 without added olive stone flour was analyzed by MALDI ToF. The results are shown in Figure 8, and their interpretation is in Table 4. First, due to the foaming process of the mixture under acid-catalyzed condition, it is inevitable that the self-condensation reaction of furfuryl alcohol does occur [42]. This can be observed from peak at 360.5 Da, which is interpreted as the condensation product of four molecules of furfuryl alcohol with Na^+^, namely, 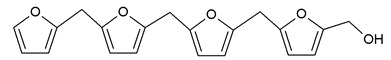


Obviously, such substances can further react with glyoxal to form furanic-glyoxal polymers, thus constituting a foam structure. For example, the peak at 557 Da can be assigned to. 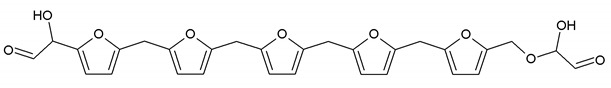


In this oligomer, the predominant reaction occurs between furfuryl alcohol and glyoxal. The peak at 232 Da is an oligomer formed by the condensation of one molecule of furfuryl alcohol with two of glyoxal, and one of the aldehyde groups combines with water to form hydrates [43]. Its reaction scheme can be 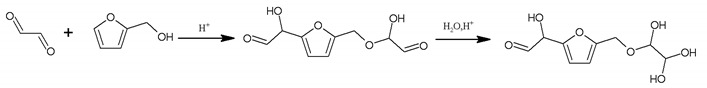


Many of these small molecule furan-glyoxal species continue to undergo condensation reactions to obtain oligomers of larger molecular weights and branched structures, as shown by the species at 800 Da: 
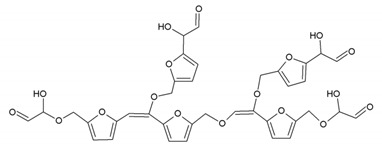


Species presenting even larger molecular weights can be observed in the MALDI ToF spectra (Figure 8), and their assignments are shown in Table 4. All these branched polymers combine to form a three-dimensional network, thus must form a foam cell wall with a certain strength after hardening to ensure compression performance.

An alternative interpretation may also be a possibility for the peak at 232 Da, which is due to a linear oligomerization of the glyoxal by aldol condensation [25]:
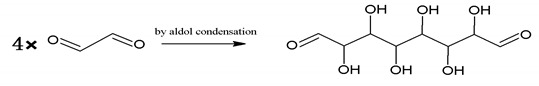


Furthermore, the aldehyde formed by aldol condensation produces more hydroxyl groups, which is also one of the reasons why there are stronger hydroxyl absorption peak in foam F3 when this is analyzed by FTIR. This kind of aldehyde can also participate in the reaction with furfuryl alcohol to contribute to the three-dimensional furanic-glyoxal structures, just like glyoxal.

## 4. Conclusions

As a biosourced chemical raw material, furfuryl alcohol was used to react with glyoxal to prepare rigid furan-glyoxal foams with uniform structures. These are self-blowing foams, with p-toluenesulfonic acid used as catalyst, the foaming process being completed at room temperature. The experimental results showed that different molar ratios of furfuryl alcohol/glyoxal had a significant effect on the foam cells morphology, which in turn affected the foams water absorption. With the reduction of furfuryl alcohol/glyoxal addition ratio, the foams gradually changed from a predominantly open-cell structure to a predominantly closed-cell one, and consequently their water absorption decreased. However, a higher furfuryl alcohol addition can provide better compression resistance to the foam. In addition, the foams so obtained have low density, poor flammability, and thus are not easy to ignite, so that they can be used as ideal wall insulation materials.

## Figures and Tables

**Table 1:** Formulations for preparing the furanic-glyoxal foams.

No.	Furfuryl Alcohol (g)	Glyoxal (g)	Olive Powder (g)	p-TSA (g)	Blowing Agent (g)
F1 (4.1)	14.7	11.6	4	3.6	1.5
F2 (4.0)	14.7	14.5	4	3.6	1.5
F3 (4.2)	11.76	14.5	4	3.6	1.5

**Table 2:** Performance of the furanic-glyoxal foams.

No.	*T*_f_/℃	*t*_p_/min	Density (kg/m^3^)	2 h Water Absorption (%)	Cell Diameter (μm)
F1	75 ± 2	7	61.1 ± 3	352.6	758 ± 15
F2	65 ± 2	10	69.1 ± 2	279.3	850 ± 20
F3	57 ± 2	11	61.7 ± 3	332.4	795.3 ± 15

**Table 3:** Combustibility characteristic parameters of furanic-glyoxal foams.

Combustion Properties	F1	F2	F3
*T*_i_ (°C)	197.22	197.08	201.52
*T*_max_ (°C)	567.67	578.33	539.33
*T*_f_ (°C)	625	647.33	615.00
*DTG*_max_ (% °C^−1^)	0.090	0.089	0.082
*DTG*_mean_ (% °C^−1^)	4.55	4.33	4.70
*S* (%^2^ K^−3^ min^−2^) × 10^−7^	0.1687	0.153	0.154

**Table 4:** Proposed oligomer structures for the MALDI ToF spectra peaks in Figure 8 for the furanic-glyoxal foams.

156 Da= Furfuryl alcohol-Glyoxal 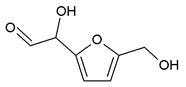
232 Da= Glyoxal-Furfuryl alcohol-Glyoxal hydrates. One of the aldehyde groups combines with water to form hydrates. 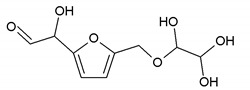
232 = 4 × glyoxal, by aldol condensation
273 Da = Glyoxal hydrates-Furfuryl alcohol-Glyoxal hydrates with Na^+^ 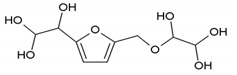
360.5 Da = 361 Da (with Na^+^) 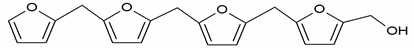
410 Da = Furfuryl alcohol-Glyoxal-Furfuryl alcohol-Glyoxal-Furfuryl alcohol 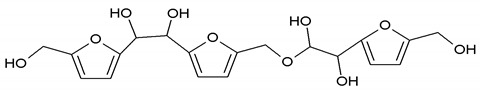
605 Da (cal 604 Da) = FA-G-FA-G-FA-G-FA-G, minus one molecule of water
623 Da = Furfuryl alcohol-Glyoxal-Furfuryl alcohol-Glyoxal-Furfuryl alcohol-Glyoxal-Furfuryl alcohol-Glyoxal
799 Da = G-FA-G-FA-G-FA-G-FA-G-FA-G, minus two molecule of water
993 Da = G-FA-G-FA-G-FA-G-FA-G-FA-G-G-FA-G, minus three molecule of water
1187 Da = G-FA-G-FA-G-FA-G-FA-G-FA-G-G-FA-G-G-FA-G, minus four molecule of water
1381 Da = G-FA-G-FA-G-FA-G-FA-G-FA-G-G-FA-G-G-FA-G-G-FA-G, minus five molecule of water
1576 Da (cal 1575Da) =G-FA-G-FA-G-FA-G-FA-G-FA-G-G-FA-G-G-FA-G-G-FA-G-G-FA-G, minus six molecule of water
1769 Da = G-FA-G-FA-G-FA-G-FA-G-FA-(-G-G-FA-G-)_5_, minus 7 molecule of water
1964 Da (cal 1963) = G-FA-G-FA-G-FA-G-FA-G-FA-(-G-G-FA-G-)_6_, minus 8 molecule of water
2158 Da (cal 2157) = G-FA-G-FA-G-FA-G-FA-G-FA-(-G-G-FA-G-)_7_, minus 9 molecule of water
2352 Da (cal 2351) = G-FA-G-FA-G-FA-G-FA-G-FA-(-G-G-FA-G-)_8_, minus 10 molecule of water
2546 Da (cal 2545) = G-FA-G-FA-G-FA-G-FA-G-FA-(-G-G-FA-G-)_9_, minus 11 molecule of water
2741 Da (cal 2739) = G-FA-G-FA-G-FA-G-FA-G-FA-(-G-G-FA-G-)_10_, minus 12 molecule of water
